# Practice of electron microscopy on nanoparticles sensitive to radiation damage: CsPbBr_3_ nanocrystals as a case study

**DOI:** 10.3389/fchem.2022.1058620

**Published:** 2022-12-20

**Authors:** Tuan M. Duong, Kshipra Sharma, Fabio Agnese, Jean-Luc Rouviere, Hanako Okuno, Stéphanie Pouget, Peter Reiss, Wai Li Ling

**Affiliations:** ^1^ Université Grenoble Alpes, CEA, CNRS, IRIG, SyMMES, STEP, Grenoble, France; ^2^ Université Grenoble Alpes, CEA, IRIG, MEM, LEMMA, Grenoble, France; ^3^ Université Grenoble Alpes, CEA, IRIG, MEM, SGX, Grenoble, France; ^4^ Université Grenoble Alpes, CEA, CNRS, IBS, Grenoble, France

**Keywords:** lead halide perovskite, low-dose electron microscopy, electron diffraction, cryo- TEM, STEM, graphene support film, HRTEM, electron beam damage

## Abstract

In-depth and reliable characterization of advanced nanoparticles is crucial for revealing the origin of their unique features and for designing novel functional materials with tailored properties. Due to their small size, characterization beyond nanometric resolution, notably, by transmission electron microscopy (TEM) and associated techniques, is essential to provide meaningful information. Nevertheless, nanoparticles, especially those containing volatile elements or organic components, are sensitive to radiation damage. Here, using CsPbBr_3_ perovskite nanocrystals as an example, strategies to preserve the native structure of radiation-sensitive nanocrystals in high-resolution electron microscopy studies are presented. Atomic-resolution images obtained using graphene support films allow for a clear comparison with simulation results, showing that most CsPbBr_3_ nanocrystals are orthorhombic. Low-dose TEM reveals faceted nanocrystals with no *in situ* formed Pb crystallites, a feature observed in previous TEM studies that has been attributed to radiation damage. Cryo-electron microscopy further delays observable effects of radiation damage. Powder electron diffraction with a hybrid pixel direct electron detector confirms the domination of orthorhombic crystals. These results emphasize the importance of optimizing TEM grid preparation and of exploiting data collection strategies that impart minimum electron dose for revealing the true structure of radiation-sensitive nanocrystals.

## Introduction

Synthetic nanoparticles, such as amphiphile assemblies, quantum dots, and metal organic frameworks, are found in a wide range of applications, including biomedicine, energy conversion, display and information technologies. Their physical and chemical characterization on the sub-nanometer scale is critical in their research and development to adapt their properties to the desired functions. Transmission electron microscopy (TEM) is a powerful and versatile tool with atomic resolution, but nanoparticles present special challenges in TEM characterization. As they very often contain light elements or volatile components (e.g., surface ligands), which transform chemically or physically under the electron beam, many nanoparticles are prone to electron beam damage during TEM studies.

Lead halide perovskite (LHP) nanocrystals present a prominent class of nanoparticles in the above category. These nanocrystals have many potential applications in optoelectronics and catalysis but they are radiation sensitive as they are highly ionic compounds with volatile elements. They have only been in the research focus since 2015, with still many unanswered questions with respect to their fundamental properties. The large surface-to-volume ratio in these nanocrystals has led to various physical properties not observed in their bulk counterparts. Most notably, at the nanoscale, LHPs exhibit narrow and tunable fluorescence emission with very high quantum yield (Schmidt et al., 2014; Protesescu et al., 2015). Coupled with a relatively facile preparation, this class of nanocrystals has become widely researched for applications in light-emitting diodes, solar cells, memristors, among other applications (Akkerman et al., 2016; Xu et al., 2017; Pan et al., 2018; Chen et al., 2020; Que et al., 2020). Structural and stability studies of these particles are indispensable to optimize their optoelectronic and spintronic properties for high-performance devices.

The crystal structure of LHP nanocrystals has been a subject of debate since the beginning and TEM has provided a lot of useful information to clarify this subject. It was initially deduced from powder X-ray diffraction studies that CsPbBr_3_ nanocrystals adopt an orthorhombic structure at room temperature although, due to the low resolution of these early studies, the cubic structure was sometimes assigned (Protesescu et al., 2015; Cottingham and Brutchey 2016). Later, advanced X-ray scattering studies raised the possibility of the existence of multiple subdomains within a single nanocrystal (Bertolotti et al., 2017). Indeed, recent TEM results show that in one single nanocrystal, the cubic and orthorhombic phases can exist simultaneously (Yu et al., 2016; Brennan et al., 2019). In the case of CsPbBr_3_ nanocubes, direct observation by TEM has been made for sizes smaller than 6 nm, which would be difficult using diffraction techniques due to the significant peak broadening for small particle sizes. Furthermore, the controlled environment of TEM experiments (i.e., high vacuum) allows the phase stability of LHP nanocrystals to be studied under the sole effect of temperature change (Zhang et al., 2019). It has been found that without the influence of pressure, the orthorhombic phase remains stable until 690 K, at which point the nanocrystals sublime. By confining the nanocrystals using amorphous carbon films, the melting and solidification processes have been directly observed, and the melting point has been determined to be 838–840 K.

Despite all the useful information provided by TEM studies, radiation damage of the LHP nanocrystals caused by the electron beam has always been a concern. In particular, the large majority of works on LHP nanocrystals with different compositions and morphologies all showed the presence of high-contrast particles of a few nanometers colocalizing with the nanocrystals (Zhu et al., 2015; Shamsi et al., 2016). These particles have been identified to be Pb nanoparticles, which have formed as a result of X-ray or electron beam irradiation of the CsPbBr_3_ crystals (Dang et al., 2017a). Strategies need to be established to avoid damage artifacts for TEM studies of the as-fabricated native structure of the LHP nanocrystals.

Recent developments in hardware, data collection strategy, and data treatment have improved our ability to image and to perform spectroscopies on challenging radiation-sensitive samples for TEM studies. Novel support films have been applied to minimize background noise. The development of direct electron detectors, including hybrid pixel detectors, has drastically reduced the electron dose necessary to obtain data with sufficient signal for analysis. The low-dose imaging technique ensures that the sample is only exposed to the required electron dose during image acquisition. Cryo-electron microscopy further reduces radiation damage by imaging at near liquid-nitrogen temperature.

Here, we examine some of the TEM grid preparation and data collection measures applied during the electron microscopy studies of lead halide perovskite nanocrystals to minimize radiation damage and to maximize the signal-to-noise ratio in data collection. We present the results from scanning TEM (STEM) and (cryo-)TEM studies of CsPbBr_3_ nanocrystals, and confront them to simulation and to published works (Yu et al., 2016; Dang et al., 2017b). The chemical and physical transformations of CsPbBr_3_ nanocrystals that can occur in electron microscopy studies are discussed emphasizing the role of the support film, which is illustrated by comparing results on amorphous carbon and graphene monolayer supports. Finally, electron and X-ray powder-diffraction are explored as tools to monitor radiation damage.

## Materials and methods

### Synthesis of CsPbBr_3_ nanocrystals

Lead bromide (PbBr_2_, >98%) was purchased from TCI Chemicals. Cesium carbonate (99.95%)*,* oleic acid (OA, 90%), oleylamine (OLA, ≥98%) and trioctylphosphine oxide (TOPO, 99%) were purchased from Sigma–Aldrich. 1- octadecene (ODE, 90%) was purchased from Acros Organics. Aluminium isopropoxide (Al(IPA)_3_, 99.99%) was purchased from Strem Chemicals. All the chemicals were used without purification.

CsPbBr_3_ core nanocrystals were synthesized by a modified method from Wu et al. (Wu et al., 2017) Briefly, 69 mg (0.188 mmol) of PbBr_2_ were mixed with 0.4 ml (1.23 mmol) OLA, 0.37 ml (1.17 mmol) OA and 328 mg (0.85 mmol) TOPO in 5 ml 1-octadecene (ODE) in a 3-neck flask in a glove box. The solution was then dried under vacuum for 1 h at 120°C before it was heated under Ar to 210°C. 0.4 ml of Cs-oleate heated to 100°C, prepared following the method of Protesescu *et al* (Protesescu et al., 2015), was injected into the flask. After 3 s, the reaction was quenched with an ice bath. The suspension was centrifuged at 7500 rpm for 6 min and the supernatant was discarded. Afterwards, 5 ml toluene was added to the precipitate and the suspension was stored under ambient air.

The synthesis typically results in nanocrystals of around 10 nm. This particle size is large enough to be resolved reliably with TEM while small enough to retain the quantum confinement effect for luminescence applications.

The synthesis of CsPbBr_3_/AlO_x_ core/shell nanocrystals was performed directly after the last step of the core synthesis. Three seconds after the injection of Cs-oleate, 333 mg of Al(IPA)_3_ in 2 ml of ODE were added to the 3-neck flask with a syringe pump (pump rate = 1 ml/min). Once the addition was finished, the reaction was quenched with an ice bath. The purification steps were identical to those used for the core nanocrystals.

### Preparation of TEM grids with graphene support film for STEM characterization

Graphene monolayers grown by chemical vapor deposition on Cu foil with a protective layer of poly-methyl methacrylate (PMMA) were purchased from Graphenea. The graphene layer was transferred onto C-flat holey carbon films (200 mesh Cu TEM grids) with hole diameter of 1.2 µm. First, the Cu substrate was etched away chemically in an aqueous solution of 0.2 M ammonium persulphate for 5 h. The layer of PMMA/graphene left floating on the solution surface was then transferred to deionized water for rinsing. After several rinsing steps, the layer was picked up by a C-flat TEM grid and air-dried. The PMMA protection layer was removed prior to sample application by immersion into acetone. Residual acetone was removed by isopropanol. The graphene grids were finally annealed at 200°C in an oxygen atmosphere for a few hours to eliminate any hydrocarbon contamination on the surface.

### STEM characterization

A sample volume of 2–4 µl was applied onto a commercial Cu TEM grid with a carbon support film (Agar scientific) or onto a graphene grid prepared as described above. Excess sample was wicked away by a filter paper. The grid with the sample was then introduced into an oven at 60°C for ∼10 min to remove hydrocarbon contaminations. STEM was performed on a probe- and aberration-corrected Thermo Fisher Themis Microscope. Images were collected at 200 kV using a high-angle annular dark field (HAADF) detector.

### TEM characterization

A sample volume of 2–4 µl was applied onto a freshly prepared Cu TEM grid covered by a homemade amorphous carbon film of nominal thickness 1.7 nm. Excess sample was removed using a filter paper. A Gatan 626 single-tilt liquid nitrogen cryo-transfer holder was used for cryo-TEM studies. The grid with the sample was either cooled to liquid nitrogen temperature in the TEM column under vacuum or plunged frozen in liquid ethane cooled by liquid nitrogen using a Thermo Fisher Vitrobot mark IV vitrification machine and cryo-transferred into the microscope. All TEM observations were performed on a Thermo Fisher Tecnai F20 cryo-TEM with a field-emission gun operating at 200 kV. Images were collected on a Thermo Fisher Ceta camera. Low-dose imaging was performed using SerialEM or Thermo Fisher EPU software.

### X-ray diffraction

X-ray diffraction measurements were performed on a Bruker D8 diffractometer equipped with a copper anode (λK_α_ = 1.54 Å) and a 1D LynxEye detector. Le Bail refinement was done with the TOPAS software.

### Electron diffraction

Electron powder diffraction patterns were collected with a selected area aperture using a Cheetah hybrid pixel direct electron detector (Amsterdam Scientific Instrument). The 2D diffraction image were azimuthally integrated to obtain 1D diffraction pattern using the free software CrysTBox (Klinger and Jager 2015). Random areas containing hundreds of particles were measured to achieve statistical significance. The evolution of the diffraction pattern with time was studied by taking consecutive images in the span of 0.5 s. The lattice parameters were calculated by using the interplanar spacings and Miller indices of the peaks (112), (220), and (004) from the diffraction pattern.

### STEM HAADF and bright field simulations

Simulation of the STEM images was performed by using the Autostem program, which implements the multislice method (Kirkland 2010). Supercells were built by using the TEM UCA server from Cadiz University (Bernal et al., 1998) and their sizes were 2.3378 nm × 2.3378 nm x 11.689 nm. The zone axes chosen for the simulations were [0 0 1] for the cubic structure and [0 1 0] and [1 0 1] for the orthorhombic crystal structure. The thickness was chosen according to an integer multiple of the unit cell parameter. Forty different thickness, starting at 3.5 nm, with a thickness step of 2.3378 nm (4 x *a*_*cubic*_ or two x *b*_*ortho*_), Cs = 0.7 mm, and 300 kV were used. In the multislice simulation, slice thickness of 0.292 nm (one atomic layer per slice) were chosen; lower thicknesses did not modify the simulated images significantly. Thirty two defocus values from −100 nm and defocus step 10 nm were used.

## Results and discussion

**FIGURE 1:**
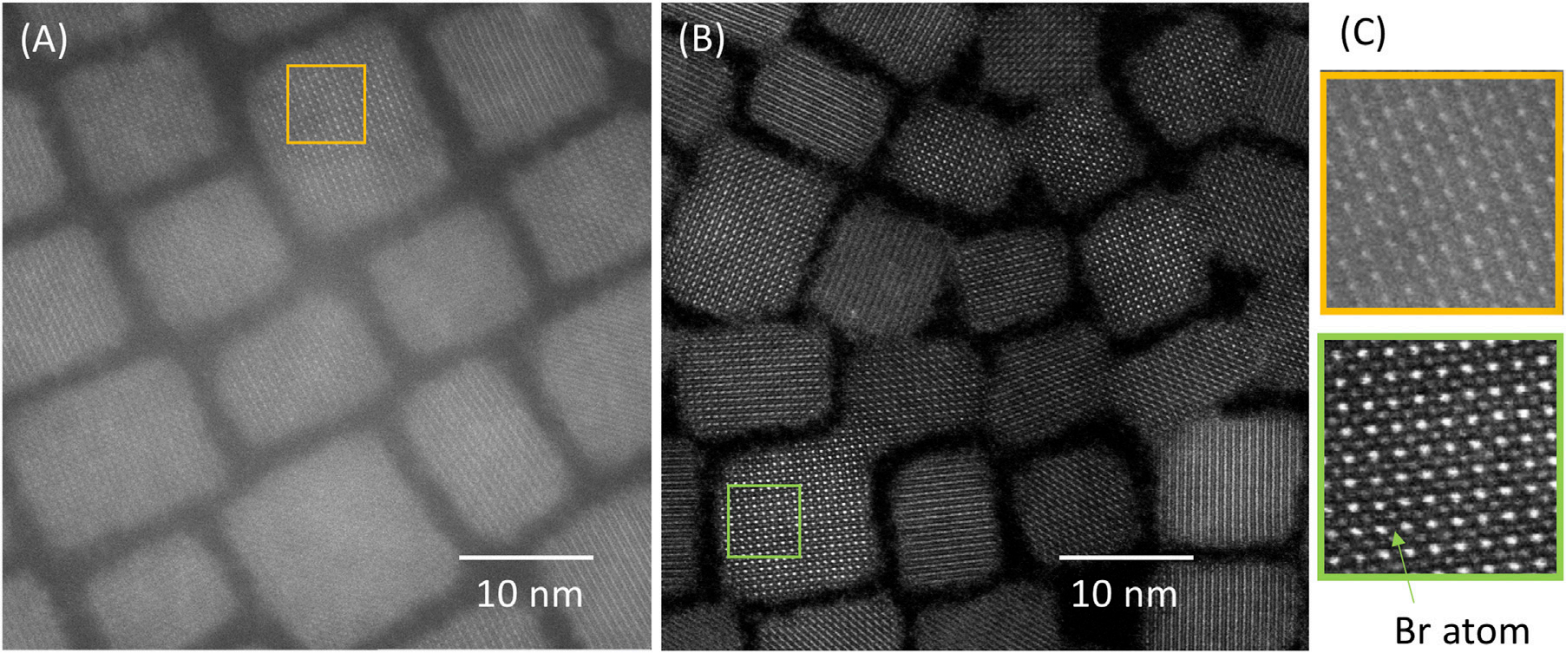
High-angle annular dark field (HAADF) scanning transmission electron microscopy (STEM) image of CsPbBr_3_ nanocrystals **(A)** on a commercial carbon film and **(B)** on transferred graphene support with the same pre-treatment for cleaning before introduction into the microscope column. **(C)** Magnified view of the boxed areas in **(A)** and **(B)**.

While solid-state samples can be suspended in vacuum for TEM observations, a support film is in general necessary for nanoparticles with all dimensions in the nanometric range. Commercial carbon films are approximately 3–10 nm thick. These films accumulate extra layers of hydrocarbon contamination if left in air. For the observation of nanocrystals in the 10 nm range (typical from LHP synthesis), scattering from commercial carbon support films significantly reduces the signal-to-noise ratio in electron microscopy studies (see Figure 1A).

**FIGURE 2:**
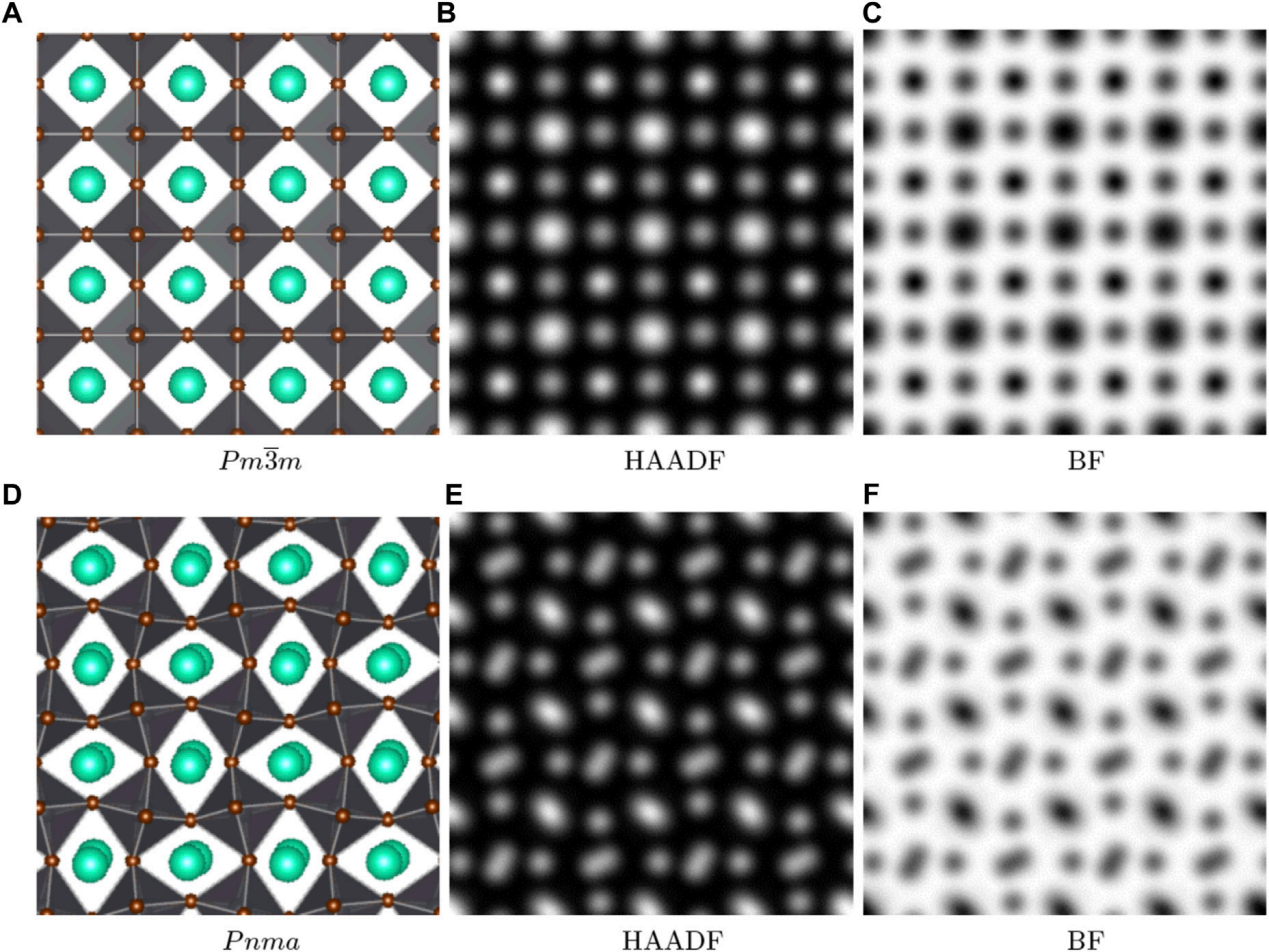
Scanning transmission electron microscopy, HAADF and bright field (BF) simulations performed on the [001] direction of the cubic structure **(A–C)** and on the [010] direction of the orthorhombic structure **(D–F)**. Cs atoms are in green, Pb in the center of the octahedra in grey, and Br in brown.

The background from the support film can be minimized by using monolayer graphene instead of carbon films. Figure 1 compares STEM images taken with a HAADF detector of CsPbBr_3_ nanocrystals supported by a commercial grid with ultra-thin carbon film (Figure 1A) and by a graphene monolayer transferred onto a C-flat grid (Figure 1B), respectively. Both samples show cuboids with rounded corners and indistinct edges. However, imaging with the sample on the graphene support film reveals clear atomic rows with high contrast, which is not the case in the sample on the amorphous carbon support. Extra atomic row projections (of Br, see simulations below) are clearly resolved in the image with the graphene support (Figure 1C and Figure 2).

The crystal structure determination of CsPbBr_3_ nanostructures has given rise to diverging reports (Brennan et al., 2019). More precisely, it has been suggested that the structure of CsPbBr_3_ nanocubes depends on their size. Small nanocubes (∼5 nm) were found to be cubic while 10 nm and larger nanocubes were assigned to the orthorhombic structure. In studies of LHP nanosheets, the cubic and orthorhombic structures have been shown to coexist (Imran et al., 2018). On the other hand, studies using pair distribution function found mostly orthorhombic structure in CsPbBr_3_ nanocubes (Cottingham and Brutchey 2016). More recently, advanced X-ray scattering studies suggested that multiple subdomains consisting of PbX_6_ octahedra titled in a cooperative manner exist in a single nanocrystal (Bertolotti et al., 2017). These works combined computer simulations with experimental data to distinguish between the two phases.

**FIGURE 3:**
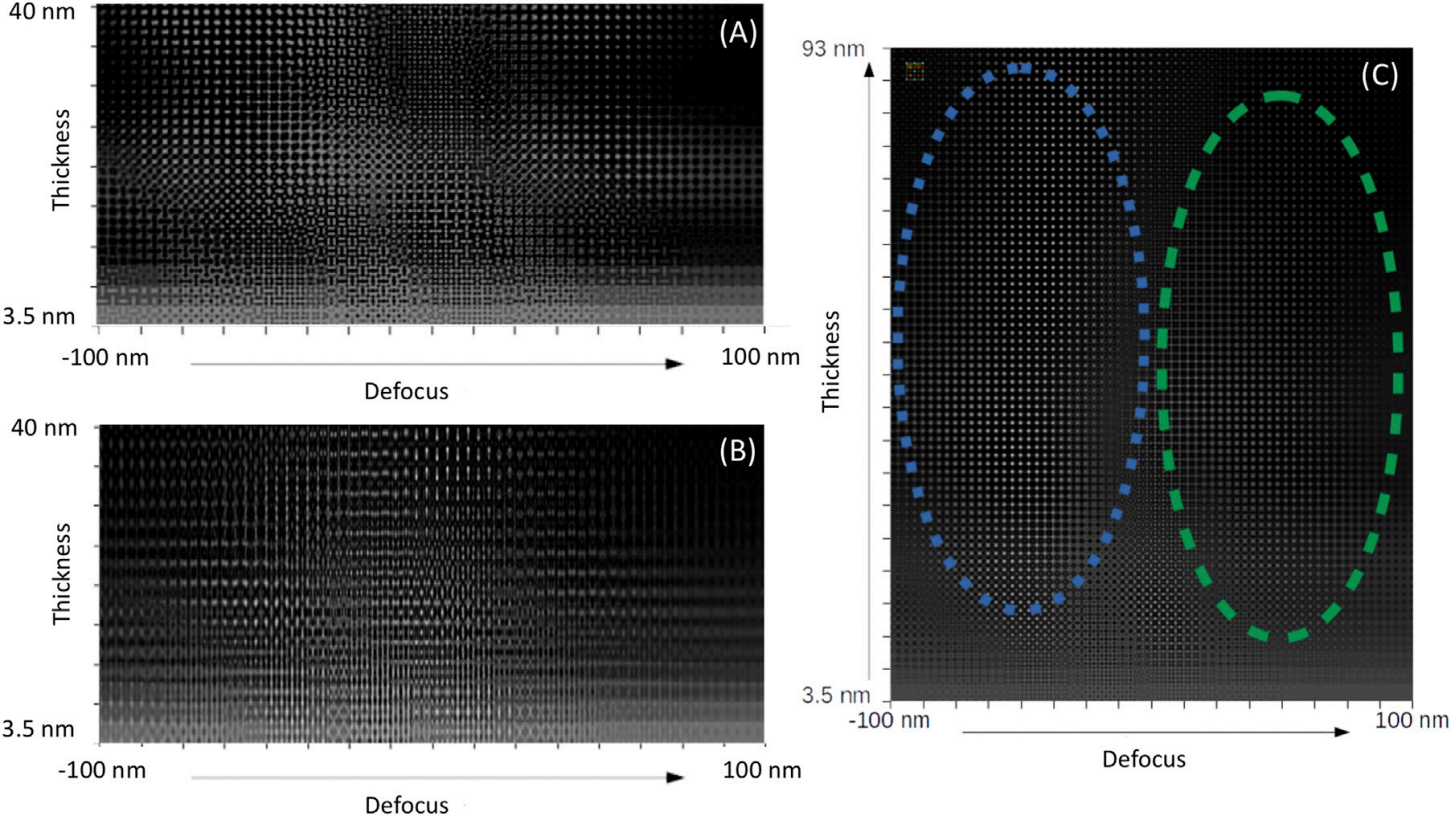
Simulated defocus-thickness high-resolution TEM (HRTEM) map. **(A)** Orthorhombic CsPbBr_3_ crystal observed along the [010] direction. **(B)** Orthorhombic CsPbBr_3_ crystal observed along the [101] direction. **(C)** Cubic CsPbBr_3_ crystal observed along the [001] direction. Atomic rows of Cs and Pb are prominent in the blue and green domains, respectively.

As seen in Figure 1, the use of a graphene support film substantially simplifies the structural studies of the CsPbBr_3_ crystal. Figures 2, 3 show our simulations performed with a sample thickness of 11.689 nm for cubic and orthorhombic CsPbBr_3_ using the unit cell parameters and atomic positions listed in Table 1. In the cubic structure, atoms of the same species are perfectly aligned along the three crystallographic axes, whereas in the orthorhombic structure, lateral displacements of some atoms are present in some projections. The fact that the atomic columns are not perfectly aligned along the zone axis gives rise to elongation in these atomic columns, the most visible difference in the simulated TEM images between the two structures. In the defocus-thickness map of the cubic phase, two large domains can be seen (marked by blue and green dotted lines in Figure 3C). In these two domains, the high-resolution (HR) TEM image looks like a simple cubic lattice. In the left domain extending roughly over 20 nm–95 nm in thickness and −70 nm–40 nm in defocus (region delineated by the blue dotted line), white spots overlap with the Cs column positions. In the right domain extending roughly over 20 nm–95 nm in thickness and 70 nm–160 nm in defocus (green dotted line), the Pb atomic columns are white.

**TABLE 1:** Unit cell parameters (Å) and atomic positions for CsPbBr_3_ perovskites with *Pm3¯m* (221) and with *Pnma* (62) space groups.

Pm $\bar{3}$ m		
a = b = c = 5.8445 Å; α=β=γ=90°		
Site	Wyckoff letter	Coordinates
Cs cation	a	0,0,0
Pb cation	b	$\frac{1}{2},\frac{1}{2},\frac{1}{2}$
Br anion	c	$0,\frac{1}{2},\frac{1}{2},\frac{1}{2}$ ;0, $\frac{1}{2}$ ; $\frac{1}{2},\frac{1}{2},0$
Pnma		
$a_{o}={8.2609 Å;b}_{o}=11.7650 Å;c_{o}=8.2124 Å;\alpha =\beta =\gamma =90{}^{\circ}$		
Site	Wyckoff letter	Coordinates
Cs cation	c	$x,\frac{1}{4},z;\bar{x}+\frac{1}{2},\frac{3}{4}$ , $z+\frac{1}{2}$ ; $\bar{x},\frac{3}{4},\bar{z};x+\frac{1}{2},\frac{1}{4},\bar{z}+\frac{1}{2}$
Pb cation	b	$0,0,\frac{1}{2};\frac{1}{2}0,0;0,\frac{1}{2},\frac{1}{2};\frac{1}{2},\frac{1}{2},0$
Br (1) anion	c	m, $\frac{1}{4}$ ,n; $\bar{m}+\frac{1}{2},\frac{3}{4},n+\frac{1}{2};\bar{m,}\frac{3}{4},\bar{n};$ m $+\frac{1}{2},\frac{1}{4},\bar{n}+\frac{1}{2}$
Br (2) anion	d	$\pm \left[u,v,w;\bar{u}+\frac{1}{2},\bar{v},w+\frac{1}{2};\bar{u},v+\frac{1}{2},\bar{w};u+\frac{1}{2},\bar{v}+\frac{1}{2},\bar{w}+\frac{1}{2}\right]$

**FIGURE 4:**
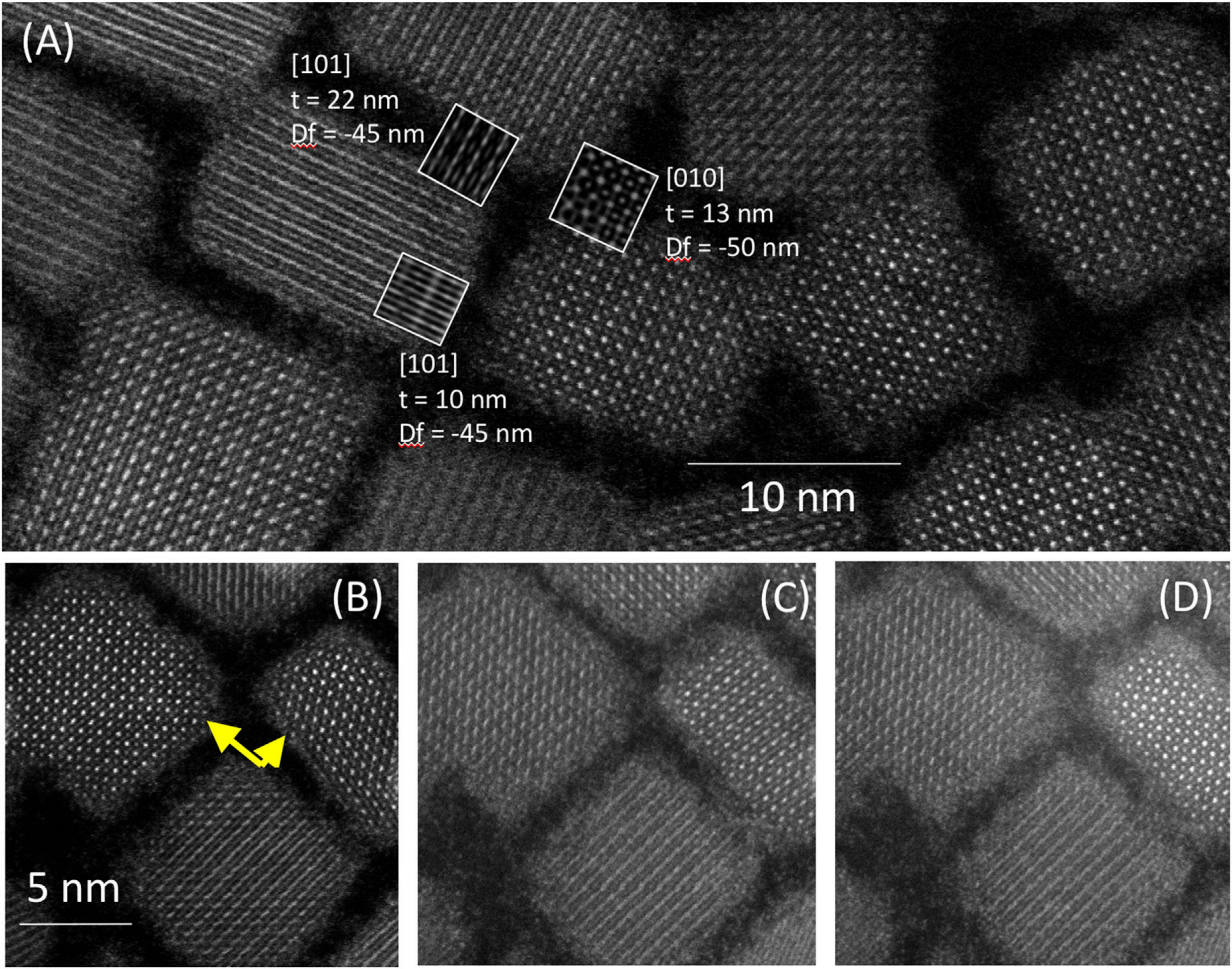
**(A)** High-resolution HAADF images of CsPbBr_3_ nanocrystals that are close to simulated projections of the orthorhombic structure. Boxed images show simulation results with various crystal orientations, crystal thicknesses along path of the electron beam (t) and defocus values (Df) for comparison. **(B–D)** Consecutive STEM HAADF scans pointing out changes in the appearance of the two nanocrystals indicated by yellow arrows from one scan to the next.

Whereas Figure 1C shows a structure that can fit the description of the cubic structure, close examination of HR images reveals other nanocrystals with structures that better fit with the projections calculated from the orthorhombic structure in Figure 3 (*cf.* Figure 4A). However, in contrast to electron diffraction (*vide infra*), this method is not appropriate to make a statistical analysis on a large number of particles due to its time-consuming character and possible structural modifications induced by the electron beam. We indeed observed changes in the imaged structure in some nanocrystals between scanned images (Figure 4B). Even though we cannot fully exclude the possibility that these differences in appearance were due to a change in the height of the nanocrystals with respect to the focal plane of the microscope, most of our observations rather point towards an electron-beam induced structural change.

**FIGURE 5:**
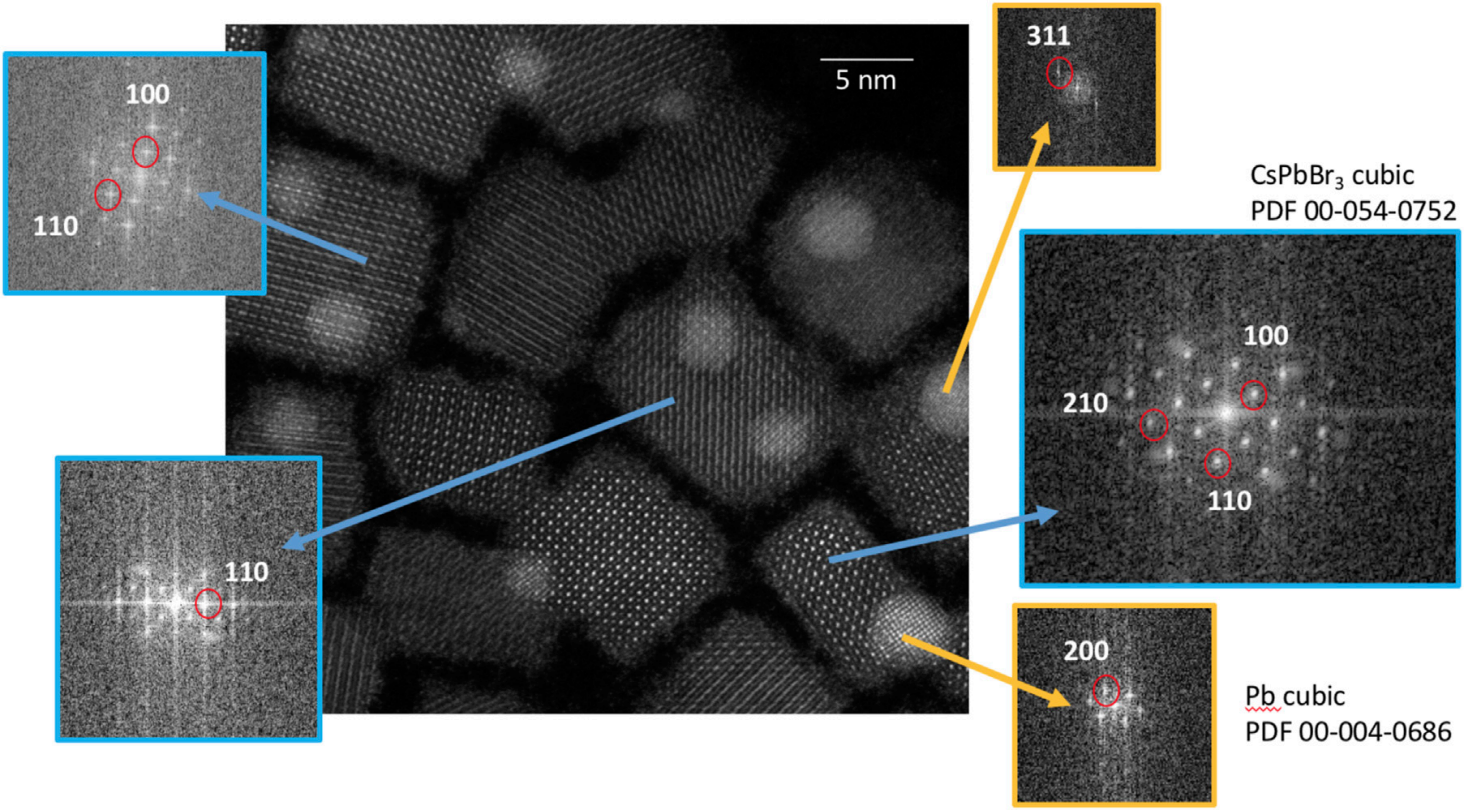
Scanning TEM image showing Pb crystallites grown on the surface of CsPbBr_3_ nanocrystals during electron beam exposure. Fourier transform images of selected areas are also shown, confirming this structural assignment.

**FIGURE 6:**
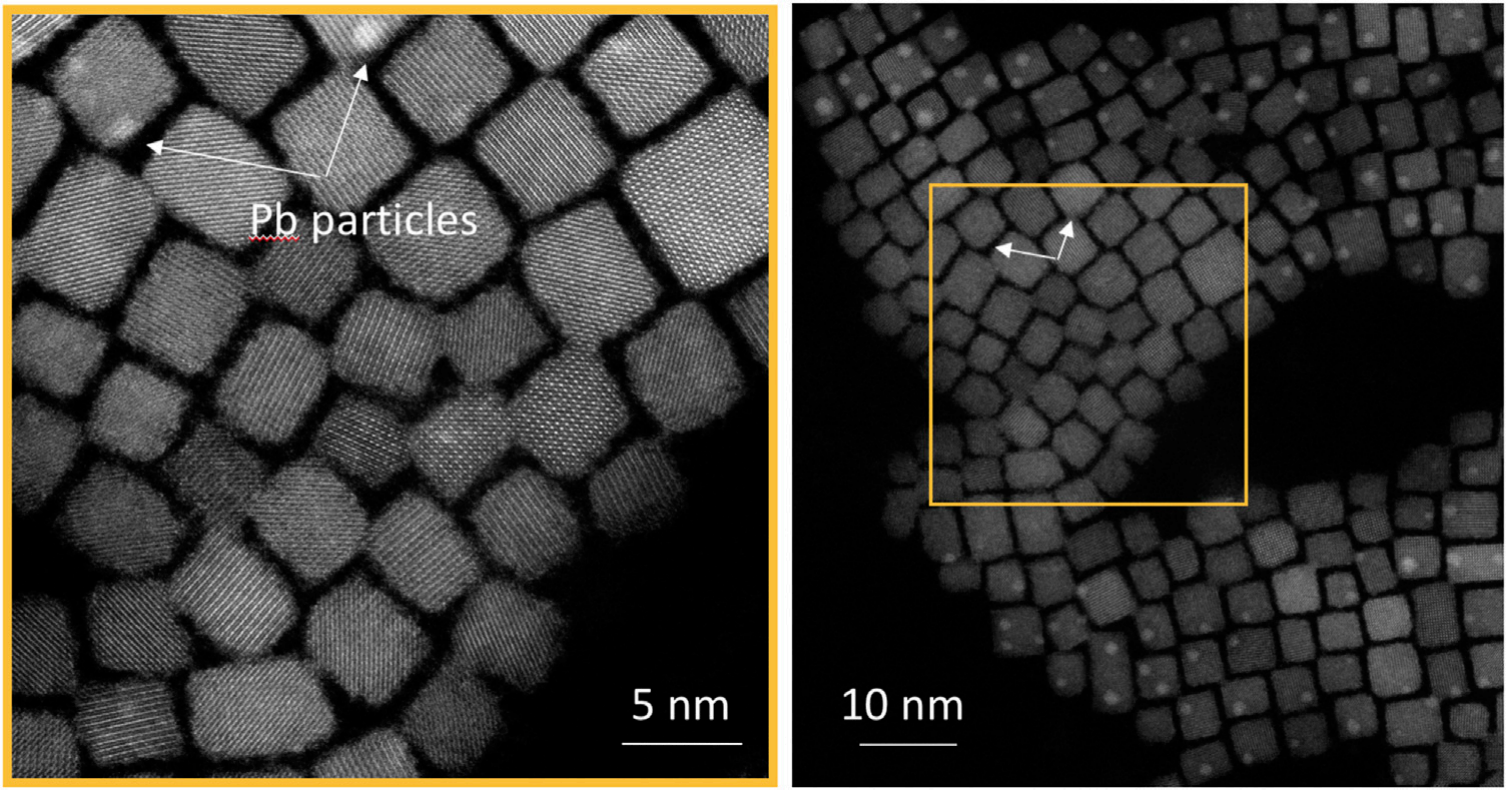
The Pb particles present in the high-magnification scan of CsPbBr_3_ nanocrystals (white arrows in the left panel) are not visible in the subsequent low-magnification image (right panel) due to their sublimation by the electron beam during the high-magnification imaging and subsequent re-condensation in neighboring areas at a lower temperature.

Corroborating this hypothesis, as seen in Figures 4B–D, there is an increasing buildup of contamination across the surface, which compromises the contrast and sharpness of the images. The organic ligand shell protecting the nanocrystals, represented by an amorphous layer at the edge of each particle, seemingly extends with each subsequent scan. This “extension” and the buildup of contamination imply that the ligands gradually degrade under the electron beam and that organic matter reorganizes around the nanocrystal surface. In the literature, a further clear sign of radiation damage during TEM studies of CsPbBr_3_ nanocrystals is the increasing presence of Pb particles on their surface, visible as bright spots in dark-field images. These particles are also present in our studies. In particular, under prolonged observation of the same zones, in addition to the CsPbBr_3_ nanocubes, smaller particles with high contrast have been observed (Figures 5, 6), mostly located near the edges and corners of the nanocrystals.

In most works on LHP nanocrystals, regardless of their morphology, the presence of similar particles of a few nanometers has been reported (Zhu et al., 2015; Shamsi et al., 2016), which have been identified as Pb nanoparticles formed as a result of electron beam irradiation (Dang et al., 2017a). Irradiation causes radiolysis of surface lead and halide ions, reducing Pb^2+^ to Pb^0^ and oxidizing Br^−^ to Br^0^ or Br^+^, which escapes from the nanocrystals. The remaining Pb^0^ atoms diffuse across the surface, eventually nucleating Pb nanoparticles on the LHP nanocrystals.

As shown in Figure 5, our observations confirm that these Pb particles grow epitaxially on the CsPbBr_3_ nanocubes with lattice spacings corresponding to those of cubic Pb crystals. As the edges and corners of the LHP nanocrystals contain a high amount of unsaturated dangling bonds, they are more severely affected by radiolysis than the atoms on the crystal facets, which explains the position of the Pb nanoparticles relative to the CsPbBr_3_ nanocrystals.

Previous studies have shown that, as irradiation continues, the Pb crystallites gradually become amorphous and dissolve (Dang et al., 2017b). The dissolved Pb^0^ atoms are distributed onto the nanocrystal surfaces as well as the supporting film of the TEM grid. In our experiments, Pb crystallites were sometimes absent in HR-STEM images. However, after the image acquisition, Pb crystallites were observed just around the border of the previously observed zone (Figure 6), indicating that Pb atoms have certainly been sublimed by the electron beam at high dose and condensed in the surroundings of the HR-scanned area.

We next compared the STEM results with TEM imaging obtained with the low-dose technique. This technique is the standard image acquisition technique for biological samples, which are notoriously radiation sensitive. With low-dose imaging, the area of interest is identified in low magnification (e.g., 1700 X with spot 8) when the electron beam is spread over a large area. The resulting electron dose imparted on the imaged region in this case is beyond the measurable dose limit (nominally 0 e^−^/Å^2^). Focusing is then performed in an area close to the region of interest without exposing the latter to the electron beam. Finally, exposure of the region of interest to significant electron irradiation occurs only when the high-resolution image of this region is acquired.

**FIGURE 7:**
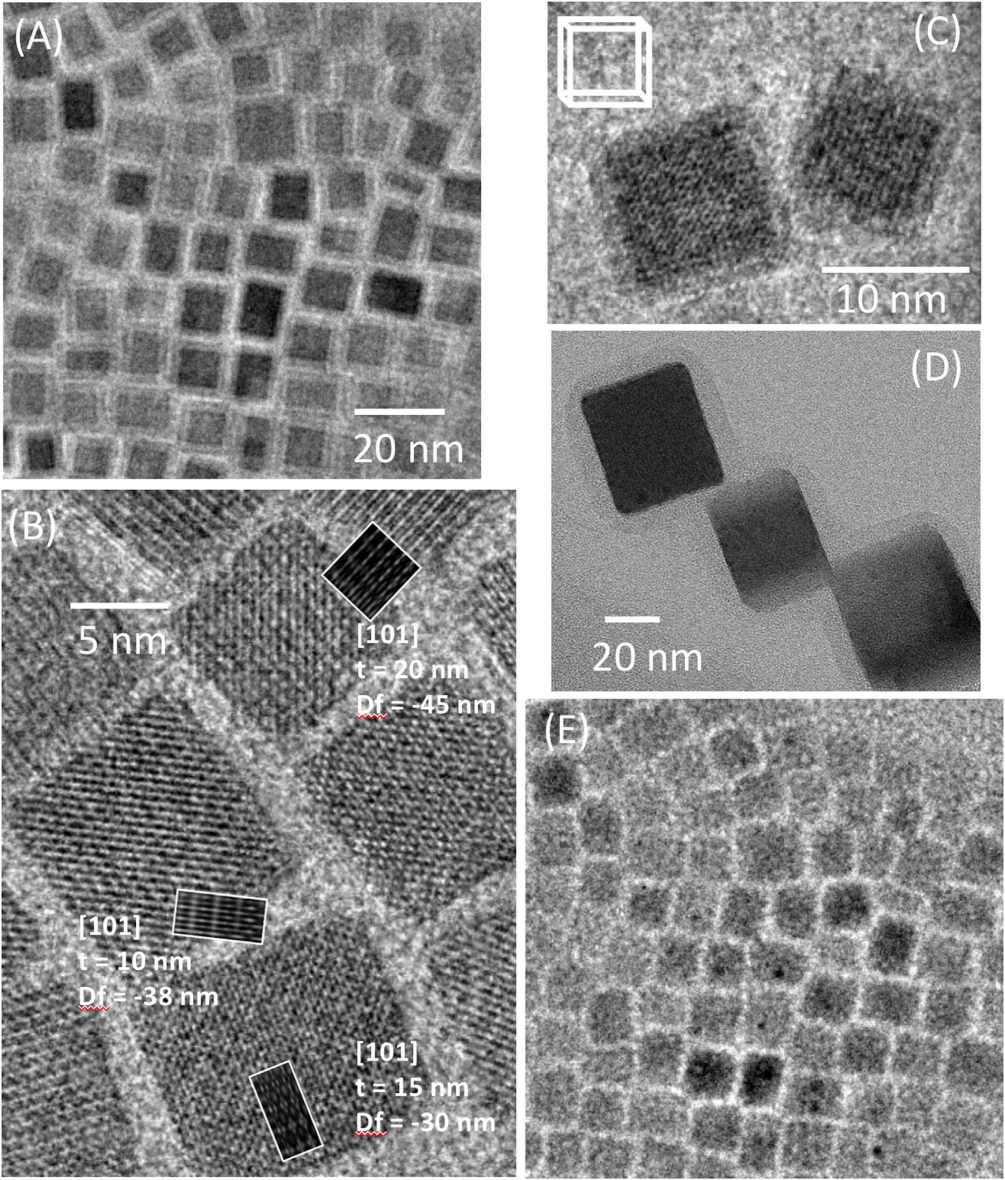
Low-dose TEM images of CsPbBr_3_ nanocrystals. **(A–C)** Pb crystallites are not observed. In **(A)**, the tilt of the nanocubes (misalignment of the crystal faces of the nanocrystals with respect to the electron beam) leads to an artificial “core/shell” structure. **(B)** High-resolution image of the same sample without tilt; no “shell” is visible. **(C)** Image of a tilted nanocuboid. **(D)** Image of CsPbBr_3_/AlO_x_ core/shell nanocrystals. **(E)** Same area as in **(A)** after repeated exposure (500 e^−^/Å^2^ total dose). Note also the appearance of Pb islands.

Images of the CsPbBr_3_ nanocrystals taken with low-dose (∼30 e^−^/Å^2^ total exposure) TEM show nanocuboids with *straight edges and sharp corners* (Figure 7A–C). Because of the well-defined edges, it is easy to discern when the alignment of the crystal faces of the nanocrystals with respect to the electron beam is not perfect, as is the case in Figure 7A. We underline that this imaging artifact can easily be interpreted in a false way as a core/shell structure. For comparison and to point out the difference between the projection of a tilted cube and that of a core/shell structure, we display in Figure 7D the image of CsPbBr_3_ nanocrystals with an amorphous alumina shell. For cuboids, we expect that the nanocrystals have the (010) and (101) planes parallel to the support film, and their 2D projection would be “perfect” rectangles. Nonetheless, misalignment can occur with a slightly tilted or bent support film, resulting in a 2D projection of the cuboid as a smaller “core” rectangle with a “shell” around it (Figure 7C inset). Since the electron beam travels through less material in the “shell” region, this region appears lower in contrast than the bulk of the nanocrystal (“core”). The effect of this artifact is reduced when the support film lies more horizontally (Figure 7B). With continued irradiation, corners of the nanocrystals rounded and the edges turned amorphous (Figure 7E). At this stage, it became difficult to tell if the electron beam was aligned with the crystallographic axis of the crystals from imaging alone. High-contrast Pb particles also started to appear as black dots.

**FIGURE 8:**
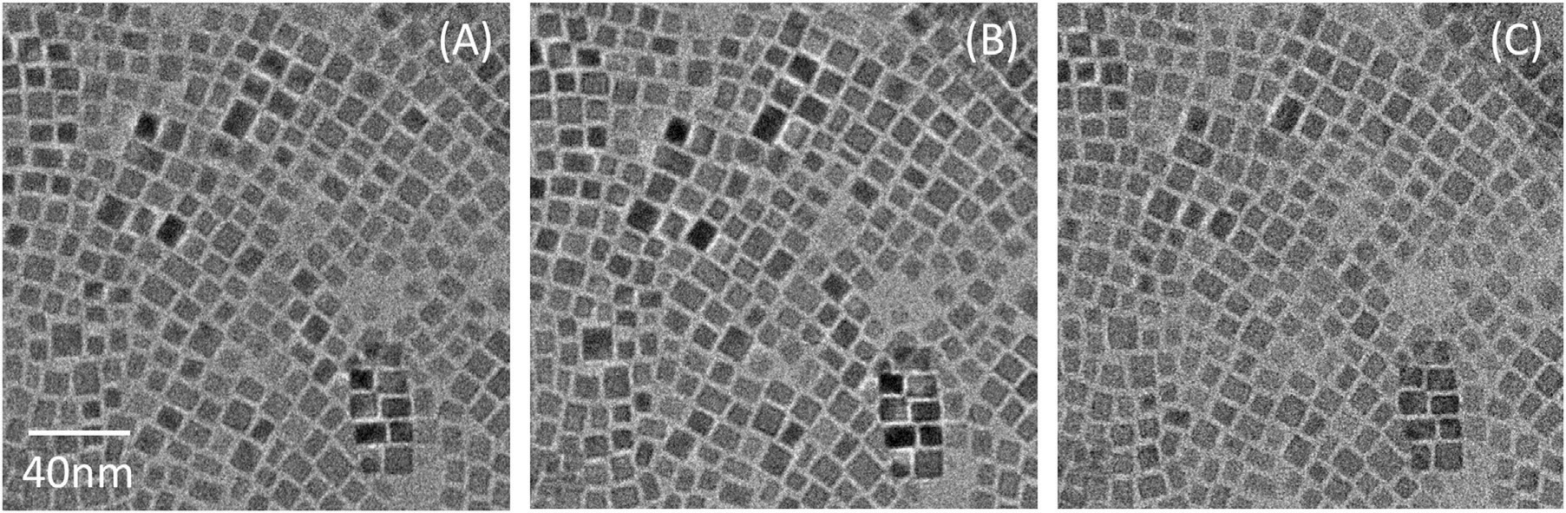
Cryo-TEM images of CsPbBr_3_ nanocrystals in slightly underfocused condition. **(A)** First exposure (46 e^−^/Å^2^) **(B)** Second exposure, which shows no noticeable difference from the first one. **(C)** 20th exposure (928 e^−^/Å^2^).

Reducing the sample temperature is a further strategy known to significantly reduce the radiation damage caused by the electron beam (Dubochet et al., 1981; Jasim et al., 2021). Moreover, because of the nature of the Pb removal and condensation process, it has been reported that Pb crystallite formation happens faster when the measurement temperature is high enough (≥−40°C) to facilitate the diffusion of Pb atoms (Dang et al., 2017a). Indeed, as we performed cryo-electron microscopy on the CsPbBr_3_ nanocrystals, no Pb island was detected after consecutive scans, supporting the assertion that Pb particle formation is inhibited at low temperature (Figure 8). The nanocrystals also retained their sharp corners and straight edges after repeated exposure to the electron beam. Thus, the crystalline structure has clearly been better preserved at cryogenic temperature than at room temperature during TEM imaging.

These cryo-electron microscopy results confirm the claims of Dang *et al.* that low temperature drastically suppresses the diffusion of Pb atoms or clusters (Dang et al., 2017b). On the other hand, it has been reported that low-temperature imaging can still induce other crystalline transformations. To elucidate this behavior, we followed the evolution of the crystal structure at room temperature and cryo-imaging conditions by using electron powder diffraction.

**FIGURE 9:**
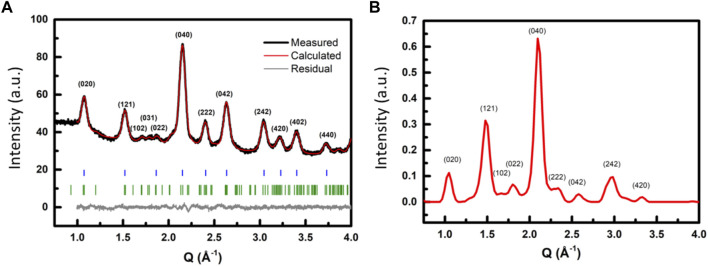
**(A)** X-ray diffractogram of the synthesized CsPbBr_3_ nanocrystals and Le Bail refinement of the data. The diffraction pattern is assigned to the orthorhombic *Pnma* space group, which differs from the cubic structure in particular by the additional peaks observed at 1.71 Å^-1^, 1.79 Å^-1^, and 2.03 Å^-1^. The blue and green bars indicate the peak positions of the cubic and orthorhombic reference patterns, respectively. **(B)** Azimuthal integration of the 2D electron diffraction pattern.

Compared to conventional X-ray diffraction, electron diffraction can probe a smaller area of interest (<10 µm) and can also complement the diffraction studies with imaging. In the case of CsPbBr_3_, the two structures of interest are distinguishable by the additional peaks in the diffraction pattern of the orthorhombic phase, namely the (201), (102) and (220), (022) peaks (ICDD #01–085–6500) (Figure 9A). Since these peaks have low intensity, the corresponding diffraction rings are not easily observed in the diffraction image. In our setup, we used a hybrid pixel detector with the Medipix^®^ technology. The detector has a high dynamic range and radiation hard properties, which allow diffraction data acquisition without the use of a beam-stopper for the central transmitted beam. The unveiled central beam allows the precise location of the center of the diffraction pattern for background subtraction and signal integration. Weak rings at high resolution could be detected using this technique. Figure 9 shows the electron powder diffraction results compared to the X-ray powder diffraction results.

With the background correction, we were able to record the (220) and (022) peaks in the electron diffraction patterns (Figure 9B), confirming the presence of nanocrystals that adopt the orthorhombic phase. From the pattern, we extracted the lattice parameters for the nanocrystals as *a* = 8.5(1) Å, *b* = 11.97(2) Å, and *c* = 8.4(1) Å. These values are in fair agreement with the values obtained from X-ray diffraction for the orthorhombic phase in the *Pnma* space group for the same nanocrystals (*a* = 8.27(2) Å, *b* = 11.69(1) Å, and *c* = 8.12(3) Å, Figure 9A). The slight difference between the values produced by the two methods stems from the lower resolution of the electron diffraction technique, as well as the small size of the crystals, which causes calculations to be less accurate. We were not able to find any area that contained only nanocrystals in the cubic phase. Measurements at 100 K were also performed, and as expected, a contraction in the lattice parameters was observed (*cf.* Table 2). The lattice parameters *b* and *c* reduced to 11.88(4) Å and 8.23(3) Å, respectively, whereas there was no significant change in the parameter *a* (8.51(3) Å). This trend is similar to that reported for bulk CsPbBr_3_ between room temperature and 4 K (Lopez et al., 2020). The anisotropic contraction is due to the more ionic nature of the Cs-Br bond compared to the Pb-Br bond. The Cs-Br bond thus experiences a stronger contraction at lower temperature, which manifests in smaller *b* and *c* parameters.

**TABLE 2:** Lattice parameters of CsPbBr_3_ nanocrystals determined from electron powder diffraction at room temperature (RT) and at 100 K (with liquid nitrogen cooling).

Lattice parameter	*a* (Å)	*b* (Å)	*c* (Å)
at 295 K (RT)	8.5(1)	11.97(2)	8.4(1)
at 100 K	8.51(3)	11.88(4)	8.23(3)

We followed the powder diffraction pattern for >100 times the exposure time (equivalent to ∼10^3^ e^−^/Å^2^) and did not observe any changes in the diffraction pattern at low temperature (cooled by liquid nitrogen). No change in the peak positions was observed at room temperature neither but high-resolution diffraction rings were fading with a cumulated dose of 10^3^ e^−^/Å^2^.

## Conclusion

We performed the in-depth characterization of CsPbBr_3_ nanocrystals using STEM and TEM techniques with a special focus on the identification of electron-beam induced structural changes. Due to the small size of the nanocrystals, the use of an electron transparent support film is crucial for high-resolution imaging as demonstrated by using a graphene support film, which yields superior image quality compared to amorphous carbon films. We found that most of the ∼10 nm CsPbBr_3_ nanocuboids exhibit the orthorhombic phase by comparing HR images with simulated images. Nonetheless, the presence of the cubic phase in a small population of the nanocrystals has also been detected. Pb islands formed by electron beam induced radiolysis of surface Pb and Br occur in STEM imaging but are not observed in low-dose TEM observations, especially for imaging performed at near liquid nitrogen temperature. Moreover, under standard imaging conditions, the corners of CsPbBr_3_ nanocrystals are becoming round and the edges amorphous, making it more difficult, for example, to distinguish between imaging artifacts and core/shell structure. These observations underline the importance of using low dose for imaging radiation sensitive samples such as LHP nanostructures. We showed that using a sensitive direct electron detector and the low-dose imaging technique can minimize the beam exposure of the sample necessary for data collection while maintaining a sufficient signal-to-noise ratio. We also demonstrated that low-temperature imaging using cryo-microscopy preserved the structure and shape of the nanocrystals and allowed their imaging for longer duration. Finally, electron powder diffraction also allows for monitoring the evolution of the crystal structure with exposure to the electron beam. This technique enables sampling a larger population of nanocrystals yielding an improved statistical analysis and the results can be directly compared with powder X-ray diffraction.

In summary, several precautions need to be taken when imaging beam-sensitive materials like LHP nanocrystals with the TEM. To make sure that the samples retain their native state, the minimal dose that gives sufficient signal-to-noise ratio should be applied, ideally in combination with cryogenic temperatures.

## Data Availability

The raw data supporting the conclusions of this article will be made available by the authors, without undue reservation.
